# Redescription of *Emplectonema viride* – a ubiquitous intertidal hoplonemertean found along the West Coast of North America

**DOI:** 10.3897/zookeys.1031.59361

**Published:** 2021-04-14

**Authors:** Cecili B. Mendes, Paul Delaney, James M. Turbeville, Terra Hiebert, Svetlana Maslakova

**Affiliations:** 1 Laboratório de Diversidade Genômica, Departamento de Genética e Biologia Evolutiva, Instituto de Biociências, Universidade de São Paulo, SP, Brazil University of Oregon Charleston United States of America; 2 Oregon Institute of Marine Biology, University of Oregon, Charleston, OR, USA Universidade de São Paulo São Paulo Brazil; 3 Department of Biology, Virginia Commonwealth University, Richmond, VA, USA Virginia Commonwealth University Richmond United States of America; 4 Department of Biology, University of Oregon, Eugene, OR, USA University of Oregon Eugene United States of America

**Keywords:** Cryptic species, marine diversity, Nemertea, species delimitation

## Abstract

*Emplectonema viride* Stimpson, 1857, a barnacle predator, is one of the most common and conspicuous intertidal nemerteans found along the West Coast of North America from Alaska to California, but it is currently referred to by the wrong name. Briefly described without designation of type material or illustrations, the species was synonymized with the Atlantic look-alike, *Emplectonema gracile* (Johnston, 1837) by Coe. Here we present morphological and molecular evidence that *E. viride* is distinct from *E. gracile*. The two species exhibit differences in color of live specimens and egg size and are clearly differentiated with species delimitation analyses based on sequences of the partial regions of the 16S rRNA and cytochrome *c* oxidase subunit I genes. In order to improve nomenclatural stability, we re-describe *E. viride* based on specimens from the southern coast of Oregon and discuss which species should be the type species of the genus. *Emplectonema viride* was one of the two species originally included in the genus *Emplectonema* Stimpson, 1857, but subsequent synonymization of *E. viride* with *E. gracile* resulted in acceptance of the Atlantic species, *E. gracile*, as the type species of the genus. We resurrect *E. viride* Stimpson, 1857 and following Corrêa’s designation, this should be the type species of the genus *Emplectonema*.

## Introduction

The genus *Emplectonema* was established by Stimpson (1857) for two species: a European species *Borlasia camillea* Quatrefages, 1846 and the newly described *Emplectonema viride* Stimpson, 1857 from the Pacific coast of North America (San Francisco Bay, CA). Stimpson did not specify the type species.

The original description of *E. viride* is but a few lines in Latin: “Corpus depressum, lineare v. proteum, supra viride, subtis album. Caput subdiscretum, marginibus albis; foveis elongatis bipartitis; fronte emarginata. Ocellorum acervi quattuor; posteriores distincti, rotundati, ocellis confertis; anteriores marginales juxta foveas, ocellis sparsis. Long. 11; lat. 0.05 poll. *Hab.* In portu ‘San Francisco;’ littoralis inter lapillos” (Stimpson 1857: 163). It lacks illustrations, apparently owing to loss of Stimpson’s plates and material during the great Chicago Fire (Griffin 1898). Griffin (1898), in a posthumously published paper, re-described *E. viride* based on the material collected during his expeditions to the coast of Alaska and Puget Sound. He characterized the species both internally and externally, provided a drawing of the stylet apparatus, and noted that the specimens from Alaska showed a darker color dorsally. He also noted that *E. viride* differs from its Atlantic counterpart *Emplectonema gracile* (Johnston, 1837), by darker body color and “narrower head with sharply defined color patterns” (Griffin 1898). Coe, in his 1901 monograph describing nemerteans from the Harriman Alaska Expedition, synonymized *E. viride* with *E. gracile*. He did not cite Griffin’s work (1989) and was apparently unaware of it. Griffin succumbed to pneumonia at the age of 26, shortly before receiving his Doctoral degree from Columbia University.

*Emplectonema gracile* was first described as *Nemertes gracile* by Johnston (1837) from the Berwick Bay in the North Sea, and it was later included in the genus *Emplectonema* by Coe (1901), as a senior synonym of *E. viride*. Corrêa (1955), in her revision of the genus *Emplectonema*, specified *E. gracile* as the type species of the genus, citing priority. Since then, *Emplectonema gracile* (Johnston, 1837) has been treated as the type species of the genus (Gibson 1995), and all green *Emplectonema* specimens with a curved central stylet and a slender elongated basis are called by that name, regardless of geographic location.

The species currently recognized as *E. gracile* is listed as having a wide geographic distribution in the Northern Hemisphere, including Japan (Hokkaido), Russia (Kamchatka Peninsula), the Aleutian Islands, the Atlantic and Pacific coast of North America, northern coasts of Europe, Mediterranean, the Romanian coast of the Black Sea, and Madeira (Gibson 1995; Turbeville 2011; Maslakova, Delaney and Turbeville unpublished observations). This species is commonly found in great numbers, often with individuals intertwined, among barnacles and mussels in natural and anthropogenic environments, where it feeds upon acorn barnacles.

Here we present molecular and morphological evidence that *E. viride* is a separate species from *E. gracile*. We compare the two cryptic species and re-describe *E. viride*, the type species of the genus *Emplectonema*, as designated by Corrêa (1955).

## Materials and methods

### Sampling

Clusters of acorn barnacles, typically *Balanus glandula* Darwin, 1854, were collected from intertidal zones at two locations in southern Oregon (Oregon Scientific Take Permits #22780 and 23609) in 2019 and 2020 by C. Mendes and S. Maslakova (Table 1, Suppl. material 1). Some of the worms were removed from the barnacles in the field, but others were entangled, so barnacles were taken to the laboratory, placed in trays, and covered with seawater until the worms crawled out. Worms were removed and kept in 150 ml glass dishes in a sea table with running seawater at ambient sea temperature (12–15 °C). Seven specimens were obtained from pilings near the Charleston Marina in November 2019, and one specimen from the Oregon Institute of Marine Biology (OIMB) Boathouse dock site was collected in October 2019 (Table 1, Suppl. material 1). Additional 10 specimens were collected from the same location in April 2019 (Table 1, Suppl. material 1). Specimens collected by J. Turbeville, in November 2019, from South Carolina were obtained by removing them from mats of the scorched mussel, *Brachidontes exustus* (Linnaeus, 1758), in the field or allowing them to crawl from detached mussel clumps in plastic bags, pyrex dishes, or buckets containing seawater. An additional six specimens from the same site at Pawleys Island, SC, were collected in 2013 and 2014. Specimens collected in November 2019 were shipped alive to the OIMB and kept in isolation to prevent accidental introduction. As some worms had mature gametes, the non-flow-through water from these individuals was changed regularly and treated with 10% hypochlorite before discarding.

Live worms were photographed with external flash using a Canon Eos 5D Mark III. For close ups, worms were anesthetized with a mixture of 1:1 MgCl_2_ and seawater. Anterior end and proboscis were removed, gently compressed between a glass slide and a cover slip, and photographed using a Spot 5.2 camera mounted on an Olympus BX51 equipped with DIC optics. Eggs, sperm, and larval stages were photographed similarly. The anterior region of each morphological voucher was fixed in 10% formalin, post-fixed in Bouin’s solution, and stored in 70% ethanol. The posterior region was preserved in 95% ethanol and kept at -20 °C until DNA extraction.

### DNA extraction, PCR amplification, and sequence analysis

Genomic DNA was extracted with DNEasy Blood and Tissue kit (Qiagen) following the manufacturer’s protocol. Partial regions of cytochrome *c* oxidase subunit I (COI) and 16S ribosomal DNA (16S rRNA) were amplified using the primer pairs in Table 2. Polymerase chain reactions (PCR) were carried out using GoTaq Green Master Mix (Promega) as follows: initial denaturation at 95 °C for 2 min; 35 cycles of denaturation at 95 °C for 15 or 40 sec; annealing at 45 °C (COI) or 50 °C (16S) for 40 sec, extension at 72 °C for 1 min; and final extension at 72 °C for 2 min. PCR products were purified either with Wizard SV Gel and PCR Clean-Up System (Promega) or enzymatically with the USB ExoSAP (Thermo Fisher). Purified products were sequenced in both directions using PCR primers at Sequetech DNA Inc. (Mountain View, CA) or Genewiz (South Plainfield, NJ). Sequences were trimmed to remove primer regions and low-quality ends, complementary strands proofread against each other using GeneStudioPro (GeneStudio, Inc.), and COI sequences were checked for stop codons. Resulting sequences are deposited in GenBank (Table 1, Suppl. material 1).

Consensus sequences were aligned in the online version of Mafft software v. 7 (Katoh et al. 2019). Additional GenBank sequences of *Emplectonema gracile* from European locations and *Emplectonema viride* (listed as *Emplectonema* sp. 1) from Oregon were included in the final alignments (Table 1, Suppl. material 1). Alignments were used as input for phylogenetic inference in RAxML v. 8.2.12 (Stamatakis 2014), as available in Cipres (Miller et al. 2012), under GTRGAMMA model with 1,000 bootstraps, and *Emplectonema buergeri* as the outgroup (GenBank accession HQ848600 and JF277616). Resulting trees (phylograms) from each dataset were used as input for PTP (Poisson tree process; Zhang et al. 2013) with default parameters. The alignments were also used as input in Automatic Barcoding Gap Discovery (ABGD) online software (Puillandre et al. 2012) with default values for all parameters. FASTA files were converted to Nexus format with PGDSpider v. 2.1.1 (Lischer and Excoffier 2012) and used as input for haplotype network constructions in PopArt v. 1.7 (Leigh and Bryant 2015) using the TCS (Templeton et al. 1992) algorithm. Datasets used in Popart were trimmed to the length of the shortest sequence to avoid biases.

## Results

### Species delimitation analysis

**Figure 1.**
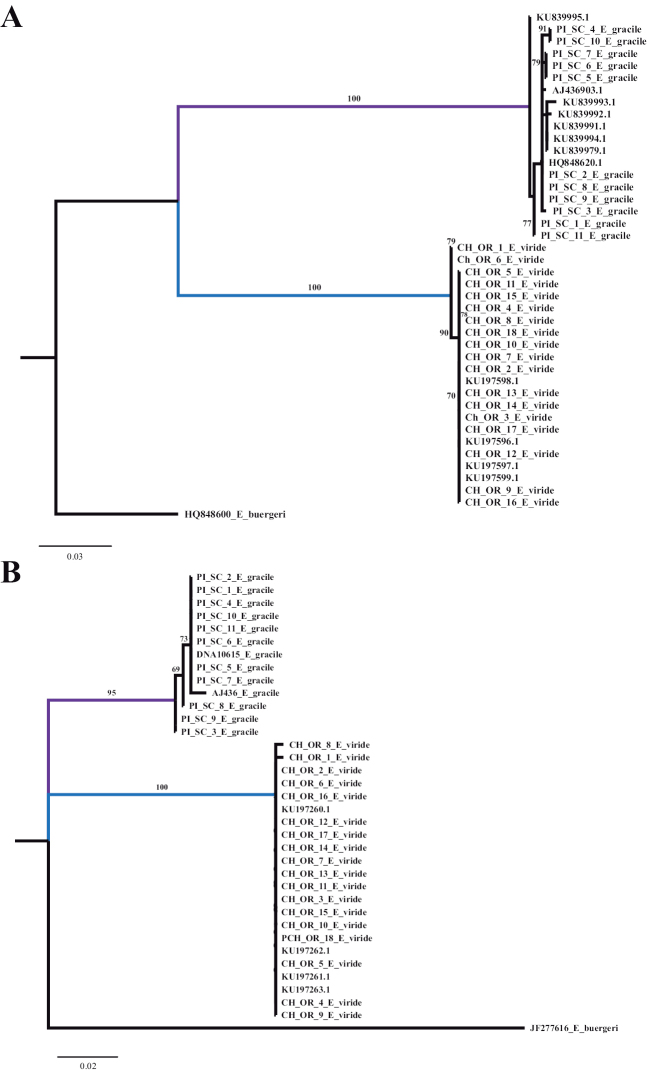
Resulting trees from the Maximum Likelihood analysis with RAxML. A: COI phylogeny (lnL = -1520.573862). B: 16S rRNA phylogeny (lnL = -909.477668). Support values above 50 presented in each branch. Branch in purple comprises specimens of *Emplectonema gracile*. Branch in blue comprises specimens of *Emplectonema viride*.

Independent phylogenetic analyses from each gene region apportioned the specimens into two main clades with high support, corresponding to sampling location. Specimens from the Pacific Ocean (*Emplectonema viride*) form one clade, and specimens from the Atlantic Ocean and North Sea (*Emplectonema gracile*) form another (Fig. 1). Results from the PTP analysis using the maximum likelihood search indicate that these two clades represent a single species each (Fig. 1, Suppl. material 2). The result from the Bayesian solution presents each specimen of *E. viride* as a different species (results not shown). These trees, however, do not show any signs of geographic influence, with specimens from the North Sea distributed among the specimens from the Atlantic coast of North America. ABGD analysis of the COI sequences found a barcoding gap at K2P distance of 0.01–0.16, while analysis of the 16S rRNA sequences found a gap at K2P distance of 0.015–0.10. Both datasets delimit the same two groups found by the phylogenetic analysis.

**Figure 2.**
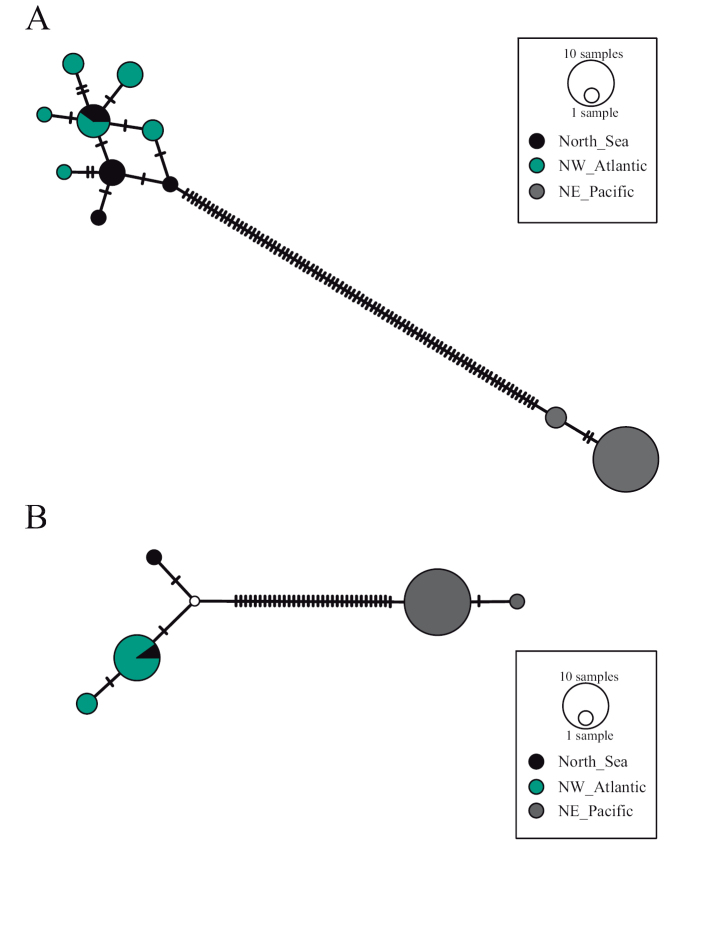
Haplotype networks of *Emplectonema gracile* (North Sea and NW Atlantic) and *Emplectonema viride* (NE Pacific) **A** generated from sequences of COI gene region **B** generated from sequences of 16S rRNA gene region.

The haplotype networks show a low diversity with many mutational steps (85 for COI and 33 for 16S rRNA) between specimens from the Pacific and Atlantic Oceans (Fig. 2). Specimens of *E. viride* comprise only two haplotypes, in both networks, with one dominant haplotype. The specimens of *E. gracile* comprise nine haplotypes, with only one shared between the Atlantic and the North Sea, and no dominant haplotype for COI sequences. 16S rRNA sequences of *E. gracile* comprise three haplotypes, with one dominant haplotype shared between locations.

### Taxonomy

#### Class HOPLONEMERTEA Hubrecht, 1879

**Order Monostilifera Brinkmann, 1917**

**Suborder Eumonostilifera Chernyshev, 2003**

**Family Emplectonematidae Bürger, 1904**

##### Genus Emplectonema

Taxon classificationAnimaliaMonostiliferaEmplectonematidae

Stimpson, 1857

3E0FD206-34EA-5CFF-A8F5-BDB8447D3EF0

###### Type species.

*Emplectonema viride* Stimpson, 1857: 163; Griffin 1898: 207.

*Emplectonema gracile*Coe 1901: 23, fig. 3; Coe 1904: 23, fig. 3; Coe 1905: 207–208, pl. 1, figs 14, 14a, 15, 15a, tex fig. 32; Coe 1940: 252, 278–280, pl. 30, fig. 40; Corrêa 1964: 517–518, 534–536; Kozloff 1999: 98, 100; Roe et al. 2007: 229, 232 pl. 89I.

###### Material examined.

Seven adults from Charleston Marina, OR (43°20.63'N, 124°19.38'W); 27 Nov. 2019; collected from wooden pilings among acorn barnacles, *Balanus glandula* (Table 1, Suppl. material 1). One specimen from OIMB Boathouse dock, OR (43°20.96'N, 124°19.80'W); 10 Oct. 2019; collected from concrete pilings among acorn barnacles, *Balanus glandula* (Table 1, Suppl. material 1). Eggs measured from two specimens collected from the jetty at the north end of Bastendorff Beach, OR (43°21.13'N, 124°20.66'W) on 29 Jan. 2020; and sperm from one male collected at Charleston Marina on 31 Jan. 2020. Voucher material is deposited at the Smithsonian Institution’s National Museum of Natural History, Washington, DC: USNM 1638666–USNM 1638673. Each specimen consists of a morphological voucher (anterior end fixed in formaldehyde, post-fixed in Bouin’s solution and stored in 70% ethanol) and a tissue sample for DNA extraction (pieces of posterior or midbody in 95% ethanol).

###### Comparative material.

Three females and four non-sexed adults of *E. gracile* from Pawleys Island, SC (33°24.63'N, 79°7.88'W); 29 Nov. 2019; among scorched mussels, *Brachidontes exustus* on granite rocks; GenBank and NMNH accession numbers in Table 1.

**Table 1.** Sampling locations, specimen ID, and accession numbers. Morphological vouchers listed in bold. † Sequences previously available in GenBank.

Species	Abbreviation	Sampling location	NMNH #	GenBank accession COI	GenBank accession 16S rRNA
*Emplectonema viride*	CH_OR_1_E_viride	OIMB Boathouse dock, OR	–	MT649099	MT647808
	CH_OR_2_E_viride		–	MT649110	MT647809
	CH_OR_3_E_viride		–	MT649101	MT647812
	CH_OR_4_E_viride		–	MT649102	MT647811
	CH_OR_5_E_viride		–	MT649109	MT647814
	CH_OR_6_E_viride		–	MT649100	MT647815
	CH_OR_7_E_viride		–	MT649103	MT647816
	CH_OR_8_E_viride		–	MT649104	MT647807
	CH_OR_9_E_viride		–	MT649105	MT647818
	CH_OR_10_E_viride		–	MT649106	MT647817
	**CH_OR_11_E_viride**	**OIMB Boathouse dock, OR**	**USNM 1638666**	**MT649107**	**MT647813**
	**CH_OR_12_E_viride**	**Charleston Marina, OR**	**USNM 1638667**	**MT649114**	**MT647820**
	**CH_OR_13_E_viride**		**USNM 1638668**	**MT649115**	**MT647810**
	**CH_OR_14_E_viride**		**USNM 1638669**	**MT649108**	**MT647821**
	**CH_OR_15_E_viride**		**USNM 1638670**	**MT649111**	**MT647824**
	**CH_OR_16_E_viride**		**USNM 1638671**	**MT649116**	**MT647823**
	**CH_OR_17_E_viride**		**USNM 1638672**	**MT649112**	**MT647819**
	**CH_OR_18_E_viride**		**USNM 1638673**	**MT649113**	**MT647822**
	E4H2	Charleston, OR	–	KU197596†	KU197260†
	E5B5		–	KU197597†	KU197261†
	E5B6		–	KU197598†	KU197262†
	E5B7		–	KU197599†	KU197263†
*Emplectonema gracile*	PI_SC_1_E_gracile	Pawleys Island, SC	–	MT649119	MT647832
	PI_SC_2_E_gracile		–	MT649121	MT647827
	PI_SC_3_E_gracile		–	MT649127	MT647825
	PI_SC_4_E_gracile		–	MT649117	MT647830
	PI_SC_5_E_gracile		–	MT649124	MT647829
	PI_SC_6_E_gracile		–	MT649125	MT647834
	**PI_SC_7_E_gracile**	**Pawleys Island, SC**	**USNM 1638674**	**MT649126**	**MT647828**
	**PI_SC_8_E_gracile**		**USNM 1638675**	**MT649122**	**MT647835**
	**PI_SC_9_E_gracile**		**USNM 1638676**	**MT649123**	**MT647826**
	**PI_SC_10_E_gracile**		**USNM 1638677**	**MT649118**	**MT647831**
	**PI_SC_11_E_gracile**		**USNM 1638678**	**MT649120**	**MT647833**
	**PI_SC_12_E_gracile**		**USNM 1638679**	–	–
	**PI_SC_13_E_gracile**		**USNM 1638680**	–	–
	**PI_SC_14_E_gracile**		**USNM 1638681**	–	–
	–	Salcombe, UK	–	AJ436903†	AJ436793†
	DNA10615	Crosby, UK	–	HQ848620†	JF277621†
	NemBar0378	Sweden	–	KU839979†	–
	NemBar0400		–	KU839991†	–
	NemBar0401		–	KU839992†	–
	NemBar0402		–	KU839993†	–
	NemBar0403		–	KU839994†	–
	NemBar0404		–	KU839995†	–
	K21	Spain	–	KU697656†	

**Table 2.** Primer pairs utilized in this study.

Gene	Forward primer	Reverse primer	Reference
COI	HCO1490 – GGTCAACAAATCATAAAGATATTGG	LCO2198 – AAACTTCAGGGTGACCAAAAAATCA	Folmer et al. 1994
16S rRNA	16SARL – CGCCTGTTTATCAAAAACAT	16SBRH – CCGGTCTGAACTCAGATCACGT	Palumbi et al. 1991
16S rRNA		16SKr – AATAGATAGAAACCAACCTGGC	Jon Norenburg unpublished

###### Description.

**Figure 3.**
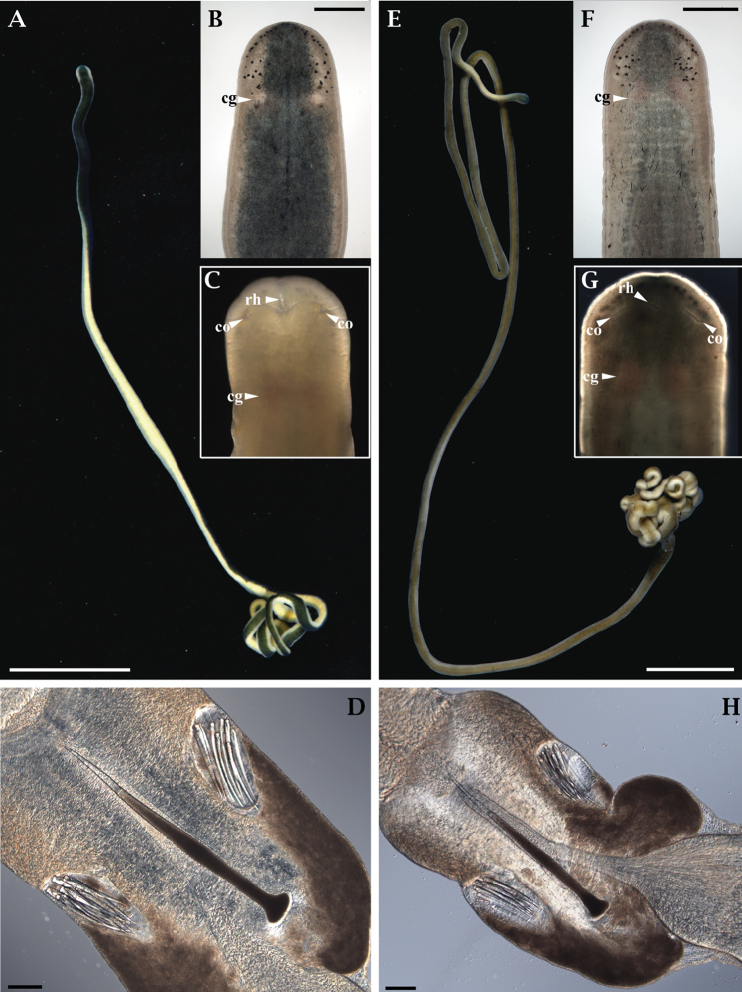
Photomicrographs of *Emplectonema viride* (**A–D**) and *Emplectonema gracile* (**E–H**). Abbreviations: cerebral ganglia, **cg**; rhynchostomopore, **rh**; cerebral organ opening, **co**. Scale bars: 10 mm (**A, E**); 0.5 mm (**B, F**); 100 μm (**D, H**).

Based on specimens from Oregon, body long and thread-like, 35–103 mm long, 0.6–1.0 mm wide. However, Griffin (1898) found specimens nearly 1 m long. Dark green dorsally, cream-colored or pale yellow ventrally (Fig. 3A). Head round, slightly wider than adjacent body when moving freely, with whitish-yellow or cream-colored margins matching the color of the ventral side (Fig. 3B). A pair of small cerebral organ furrows (anterior cephalic furrows), each shaped as a small arch, is located ventrally, anterior to cerebral ganglia (Fig. 3C). Head furrow (posterior cephalic furrow) is shaped as a dorsal posteriorly directed “V” located behind the cerebral ganglia, and only just barely noticeable in some individuals, and not detectable in many individuals. Rhynchostomopore is a small antero-ventral opening. Numerous small ocelli arranged in two groups on each side of head. Each anterior group has 8–10 eyes arranged in a narrow row along the anterior margin of the head. Each posterior group has 10–12 eyes in a dispersed cluster in front of the brain (Fig. 3B). Cerebral ganglia are pinkish and show through the body wall, especially in lighter-colored individuals. Cerebral organs are not easily distinguishable in life, but with slight compression. Posterior tip of body tapered.

**Figure 4.**
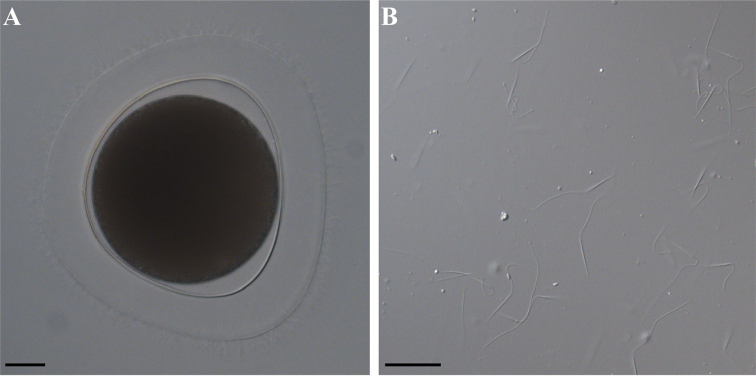
Photomicrography of egg (**A**) and sperm (**B**) of *Emplectonema viride*. Scale bars: 25 μm.

Rhynchocoel is short, approximately 1/3 of body length. Central stylet slightly curved, 170–326 μm long (*n* = 7), smooth. Basis is slender, 480–815 μm long (*n* = 7), its distal end abruptly widening into a truncated bulb (Fig. 3C). Basis length/width ratio 11.5–16.0. Basis/stylet length ratio 2.0–2.8. Two accessory stylet pouches, each with 9–13 accessory stylets (Fig. 3C). Proboscis bulb elongated. Lateral intestinal diverticula beginning at posterior of rhynchocoel, present until posterior tip of the body. Separate sexes. Gonads serially arranged between intestinal diverticula. Testes of mature males are visible through the body wall as whitish sacs. Ovaries of mature females are visible through the body wall, and the oocytes are orange to light pink, with distinct germinal vesicles. Spermatozoa with elongated head 16–20 μm. Oocytes are 110–140 μm in diameter and surrounded by a tight chorion and a jelly coat (Fig. 4).

###### Reproduction and larval development.

**Figure 5.**
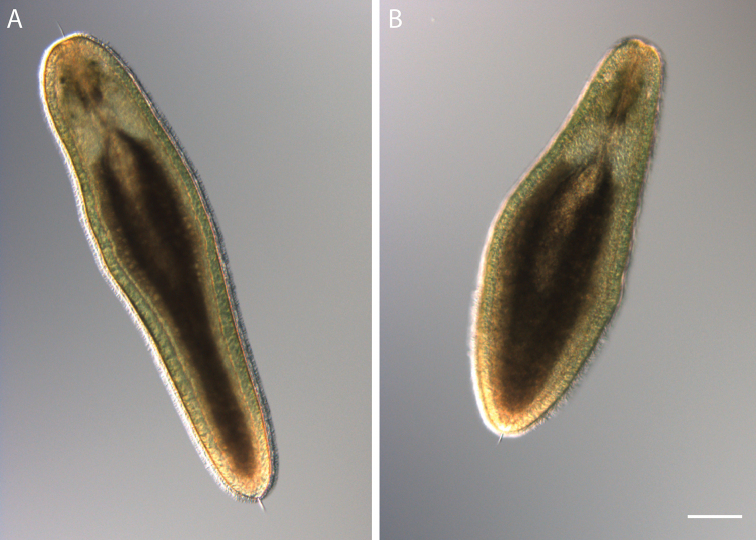
Larva of *Emplectonema viride* collected from plankton in Charleston, OR, on 17 Oct 2013. Same individual is shown in two focal planes to highlight apical tuft (upper left, **A**) and posterior cirrus (lower right, **A**) and green epidermal pigment (**B**). Note paired subepidermal eyes, which are anterior to cerebral organs. Scale bar: 100 μm.

Reproductive individuals of *E. viride* were collected in Charleston, OR, in September 2009, October 2019, January 2020, and June 2020. When ripe, males and females free-spawn gametes into the water, with no known reliable spawning cue. Swimming larvae hatch from the egg chorion after ~30 h and begin feeding on small planktonic crustaceans after developing a functional proboscis and stylet (~4 d). Planktonic period lasts several months (Mendes unpublished observations). Wild-caught larvae of *E. viride* were found in the plankton samples taken with 50–153 μm net at the Charleston Marina, OR, in October 2013, March 2019, February 2020, and June 2020. *Emplectonema viride* larvae are easily recognized by their distinctive green color (Fig. 5).

###### Distribution.

Northeastern Pacific Ocean from Alaska to California. Type locality is San Francisco Bay, California, USA.

###### Morphological comparison with *Emplectonema gracile*.

As has been pointed out by Griffin (1898), specimens of *E. viride* have a darker dorsal surface, with a distinctly lighter colored ventral side and head margins, compared to those of *E. gracile* (Fig. 3). We confirm this finding and can also add that *E. viride* has smaller eggs: 110–140 μm (*n* = 9), compared to 181–198 μm eggs of *E. gracile* (*n* = 8). The characteristics of stylet apparatus do not overtly differ in the two species (Fig. 3C, F).

## Discussion

### Differentiating cryptic species

The simple morphology of nemertean worms makes it notoriously difficult to identify species, and the use of DNA sequence data as well as gamete morphology can help differentiate between morphologically cryptic nemertean species (e.g., Strand and Sundberg 2005; Sundberg et al. 2009a, b, 2016; Chen et al. 2010; Hao et al. 2015; Hiebert and Maslakova 2015; Kajihara et al. 2018; Cherneva et al. 2019). Using mitochondrial sequence data alone can present difficulties in separating phylogenetic and biogeographical signals (Toews and Brelsford 2012 and references therein). However, analyses herein show that both mitochondrial markers exhibit signs of prolonged genetic isolation between the two species. Furthermore, all explicit, non-phylogenetic delimitation analyses in this study (ABGD and the haplotype network) show similar and well-supported results. Importantly, these tests have different strategies of species delimitation. ABGD is based on the pairwise differences between sequences. It uses an algorithm that calculates the divergence between sequences and automatically infers the barcoding gap between groups of sequences (Puillandre et al. 2012). PTP is a tree-based method that uses the number of substitution events as given by branch lengths of an input phylogram to infer intra- and interspecific relationships between sequences (Zhang et al. 2013). The TCS method used to construct haplotype networks has an integrated view of phylogeny and population structure, taking recombination into account. The algorithm collapses sequences into haplotypes, then uses the haplotype frequencies and pairwise comparison to calculate probabilities of relationship between sequences. The haplotypes are only linked when there is over 95% probability of parsimony for their connection (Templeton et al. 1992; Clement et al. 2001). These methods have been used in many delimitation studies with great success (e.g., Jörger et al. 2012; Scarpa et al. 2016; Mills et al. 2017; Pozzi et al. 2020). Therefore, congruent results using these different methods provide strong evidence for separation between *E. viride* and *E. gracile*. The morphological similarities between the two species are likely due to shared recent ancestry, but also possibly due to their similar ecology. Both species live among and prey upon barnacles. This similarity in their ecology is likely a strong factor maintaining the morphological traits even after prolonged isolation between populations (Fišer et al. 2018).

The combination of molecular and morphological data presented here confirms the existence of two cryptic species of North American *Emplectonema*, one from the Pacific and another from the Atlantic coast. Our results support the validity of *E. viride* described from the Pacific coast (Stimpson 1857; Griffin 1898) and suggest that Coe’s (1901) synonymization of *E. viride* with *E. gracile* is unjustified.

### Genus type fixation

The genus *Emplectonema* was established by Stimpson (1857) for *Emplectonema viride* and *Borlasia camillea* Quatrefages, 1846 (subsequently treated as a synonym of *Amphiporus neesii* Örsted, 1844 by McIntosh (1873–1874), Bürger (1895), and others. However, Stimpson (1857) did not designate a type species. According to the Article 67.2 of the ICZN, only the species originally included are eligible to be fixed as the type species of the genus (ICZN 1999). This makes *E. gracile* ineligible for designation as the type species of the genus. So, which species should become the type of *Emplectonema*?

DNA-based phylogenies (18S rRNA, COI) suggest that *Emplectonema neesii* (Örsted, 1844) is not closely related to *E. gracile* (Strand and Sundberg 2005; Sundberg et al. 2009b). Results of Sundberg et al. (2009b) also show a close relationship between *E. neesii* and *E. buergeri* based on COI data, a relationship also supported by morphological similarities. A more recent multi-locus phylogeny of the phylum (Andrade et al. 2011) shows that *E. gracile* is not closely related to *E. buergeri*, rendering the genus *Emplectonema* polyphyletic. Clearly, the two species originally included and eligible to be fixed as the type species of the genus *Emplectonema* should not belong to the same genus. We follow Correa’s designation of *E. gracile* as the type species of the genus. Article 69.2.2 of the Code says “If an author designates as type species a nominal species that was not originally included (or accepts another’s such designation) and if, but only if, at the same time he or she places that nominal species in synonymy with one and only one of the originally included species (as defined in Article 67.2), that act constitutes fixation of the latter species as type species of the nominal genus or subgenus” (ICZN 1999). As Corrêa (1955) followed Coe’s (1901) taxonomic view that the taxonomic species, *E. gracile* includes the nominal species *Nemertes gracilis* and *E. viride*, but not *Borlasia camillea*, the type species has been validly fixed subsequently by Corrêa (1955) as *Emplectonema viride* (Kajihara personal communication). Therefore, *E. viride* and not *A. neesii* should be the type species of *Emplectonema*. *Amphiporus neesii* is not an *Emplectonema* and is treated here as a species *incertae sedis*.

## Supplementary Material

XML Treatment for Emplectonema

## Supplementary materials

Supplementary material 1 Table S1 Data type occurrence Explanation note Linked data table of specimens and sequences analyzed in this study. Abbreviations. COI: Cytochrome oxidase I gene fragment; 16S rRNA: 16S ribosomal RNA; †: Sequences previously available in GenBank. Specimens morphologically analyzed marked in bold. https://binary.pensoft.net/file/531664This dataset is made available under the Open Database License (http://opendatacommons.org/licenses/odbl/1.0/). The Open Database License (ODbL) is a license agreement intended to allow users to freely share, modify, and use this Dataset while maintaining this same freedom for others, provided that the original source and author(s) are credited.Cecili B. Mendes, Paul Delaney, James M. Turbeville, Terra Hiebert, Svetlana Maslakova

Supplementary material 2 Figure S2 Data type phylogenetic Explanation note Species delimitation results from PTP for COI (**A**) and 16S rRNA (**B**) gene regions. Values on branches are posterior probabilities of those taxa form one species under the PTP model and a flat prior. Red branches indicate taxa that should be considered as part of the same lineage. https://binary.pensoft.net/file/531665This dataset is made available under the Open Database License (http://opendatacommons.org/licenses/odbl/1.0/). The Open Database License (ODbL) is a license agreement intended to allow users to freely share, modify, and use this Dataset while maintaining this same freedom for others, provided that the original source and author(s) are credited.Cecili B. Mendes, Paul Delaney, James M. Turbeville, Terra Hiebert, Svetlana Maslakova
